# Ginkgo biloba extract EGb 761^®^ versus pentoxifylline in chronic tinnitus: a randomized, double-blind clinical trial

**DOI:** 10.1007/s11096-018-0654-4

**Published:** 2018-06-01

**Authors:** Klára Procházková, Ivan Šejna, Jan Skutil, Aleš Hahn

**Affiliations:** 1 Department of Otorhinolaryngology, University Hospital Kralovske Vinohrady, Šrobárova 50, 10034 Prague, Czech Republic; 2 Third Faculty of Medicine, Charles University, Prague, Czech Republic

**Keywords:** Effectiveness, Ginkgo biloba, Pentoxifylline, Tinnitus

## Abstract

*Background* Ginkgo biloba extract EGb 761^®^ and pentoxifylline are frequently prescribed for the treatment of tinnitus. *Objective* To compare the treatment effects of Ginkgo biloba extract EGb 761R and pentoxifylline. *Setting* The study was performed at Department of Otorhinolaryngology of University Hospital Královské Vinohrady and 3rd Medical Faculty, Charles University in Prague. *Method* Patients with sub-chronic or chronic tinnitus were enrolled in double-blind trial and randomized to receive 120 mg EGb 761^®^ or 600 mg pentoxifylline, each twice a day and in double-dummy fashion over a 12-week period. *Main outcome measure* changes in 11-Point Box Scales for tinnitus loudness and annoyance, the abridged Tinnitus Questionnaire (Mini-TQ), the Hospital Anxiety and Depression Scale (HADS), and the Sheehan Disability Scale (SDS). *Results* Full analysis set for efficacy analysis comprised 197 patients (EGb 761^®^, 99; pentoxifylline 98). For both treatment groups, significant improvements were observed in the Mini-TQ, the 11-Point Box Scales for tinnitus loudness and annoyance, the HADS anxiety score and the SDS. There was no relevant difference with regard to tinnitus-related outcomes between the two treatment groups. 20 adverse events were documented in EGb 761^®^ group and 36 adverse events were reported for pentoxifylline group. No serious adverse event was reported during the study. *Conclusion* EGb 761^®^ and pentoxifylline were similarly effective in reducing the loudness and annoyance of tinnitus as well as overall suffering of the patients. The incidence of adverse events was lower in the EGb 761^®^ group.

## Impact on practice

- EGb 761^®^ and pentoxifylline have essentially similar efficacy.
- With lower rates of adverse events, EGb 761^®^ may be the safer choice.

## Introduction

Tinnitus is a sound perceived by the patient although there is no corresponding external source of such a sound. It represents a widespread medical problem. The aetiology of tinnitus is multiple and may vary from cochlear lesions and disturbances in hearing pathways to metabolic, cardiovascular or musculoskeletal disorders [1, 2]. Tinnitus is a major health problem, with an estimated prevalence of 9.6% [3] and a 10-year cumulative incidence rate of 12.7% in the United States [4]. In a United Kingdom economic study, the average annual healthcare cost of tinnitus was estimated at 717 GBP (828 EUR) per patient 750 million GBP (867 million EUR) for the National Health Service [5]. The treatment of tinnitus is difficult, not least due to the individual psychological reactions of the affected patients [6, 7]. Drugs used for tinnitus treatment include local anaesthetics, anti-depressants, benzodiazepines, drugs that aim at enhancing blood flow in the cochlea and brain (Ginkgo biloba extract EGb 761^®^, pentoxifylline, betahistine), drugs lowering the inflammatory reaction (prednisolone, dexamethasone) or drugs improving neuroplasticity (EGb 761^®^) [8].

## Aim of study

The objective of this randomized, double-blind, reference-controlled single-centre trial was to compare the treatment effects of Ginkgo biloba extract EGb 761^®^ and pentoxifylline in subjects with sub-chronic or chronic tinnitus focusing on psycho-social problems. The safety and tolerability of the two treatments were assessed as secondary outcome.

## Ethics approval

The study was conducted in accordance with the International Conference on Harmonisation (ICH) Good Clinical Practice (GCP) guideline [9], the Declaration of Helsinki and its later amendments, and national laws. The study protocol and conduct were approved by the independent Ethics Committee of the Královské Vinohrady University Hospital, Prague, Czech Republic. All subjects received oral and written information about the trial and gave their written informed consent before enrolment and before undergoing any study-related procedures.

## Methods

### Study design and study population

The trial was designed as a randomized, double-blind, double-dummy, parallel-group, single-centre trial at the Department of Otorhinolaryngology of the Královské Vinohrady University Hospital, Prague, Czech Republic. It was registered in the public clinical trials register ISRCTN under number 68772788.

We enrolled male and female patients aged ≥ 30 years with unilateral or bilateral chronic or subchronic tinnitus of at least 3 months’ duration. Subjects were eligible for participation if their tinnitus was maskable (by noise masking), the degree of annoyance by tinnitus was rated at least 3 on an 11-Point Box Scale (type of numeric analogue scale) at screening and baseline visits, they scored at least 5 on the abridged Tinnitus Questionnaire (Mini-TQ) [10] at baseline and had given informed consent.

Patients were excluded from the study if they had acute or chronic otitis media, vestibular neuritis or drug-induced tinnitus, if they were taking any other treatment for tinnitus, if they had severe cardiovascular, renal or hepatic disorders, malignant diseases, insulin-dependent diabetes mellitus or gastro-intestinal disorders leading to impaired drug absorption. Any drugs taken to treat tinnitus had to be discontinued at least 8 weeks (Ginkgo extracts at least 12 weeks before baseline. Patients who needed drugs that could possibly interfere with the effects of the investigational treatments (e.g. due to agonistic or antagonistic action on common pharmacodynamics pathways), who were taking anticoagulants or who were known to be allergic to the investigational drugs were also excluded. Female patients of childbearing age were only included under safe contraception.

### Randomization and interventions

The random allocation sequence was generated by the sponsor using a validated computer program matching drug numbers to treatments in a 1:1 ratio. The randomisation sequence was concealed by using identical labels and packages for both treatments with ascending drug numbers. The list matching drug numbers with treatments was unavailable to persons involved in conducting the study. Double-blinding was achieved by the double-dummy technique, i.e. all patients received the same number of tablets, either EGb 761^®^ and pentoxifylline-like placebo or pentoxifylline and EGb 761^®^-like placebo. Tablets containing active drug and the corresponding placebo tablets were indistinguishable in texture, colour, shape and size.

For the duration of 12 weeks, the subjects randomized to receive EGb 761^®^ took one film-coated tablet of 120 mg EGb 761^®^ together with one pentoxifylline-like placebo tablet twice a day; those randomized to pentoxifylline took one extended-release tablet of 600 mg pentoxifylline together with one EGb 761^®^-like placebo tablet twice a day. EGb 761^®^^1^ is a dry extract from Ginkgo biloba leaves (35–67:1), extraction solvent: acetone 60% (w/w). The extract is adjusted to 22.0–27.0% ginkgo flavonoids calculated as ginkgo flavone glycosides and 5.0–7.0% terpene lactones consisting of 2.8–3.4% ginkgolides A, B, C and 2.6–3.2% bilobalide and contains less than 5 ppm ginkgolic acids.

### Outcomes

The therapeutic effects of EGb 761^®^ and pentoxifylline were assessed using tinnitus-related rating scales as well as assessments of psychological symptoms and functioning.

Two separate 11-Point Box Scales for tinnitus loudness (extending from 0 = no tinnitus at all to 10 = extremely loud tinnitus) and annoyance by tinnitus (extending from 0 = not annoying at all to 10 = unbearably annoying) were filled in every day. The evaluation of the scales was based on mean weekly values per subject. The Mini-TQ [10] is an abridged, 12-item version of the Tinnitus Questionnaire (TQ) [11]. It was designed to reflect tinnitus-related psychological distress and to investigate the dimensions of the complaint about tinnitus such as subjective perception, coping attitudes and beliefs about tinnitus.

The Hospital Anxiety and Depression Scale (HADS) [12, 13] was designed to assess the presence and severity of mild, even sub-syndromal degrees of anxiety and depression. Since no somatic items are included, the scale is feasible to measure depression in somatic illnesses. The Sheehan Disability Scale (SDS) [14] is a brief 3-item self-rating tool, designed to measure the extent to which three major sectors in the patient’s life (work/school, social life, family life) are impaired by panic, anxiety, phobic or depressive symptoms.

Safety and tolerability of both investigational products were assessed by physical examination, otological examination, ECG measurements, laboratory tests and vital signs measurements.

### Statistical analysis

For each of the effectiveness outcomes the EGb 761^®^ group was compared to the pentoxifylline group using descriptive data analysis methods. The comparison of the treatment groups with respect to the 11-Point Box Scales and the Mini-TQ total score was performed using an analysis of covariance (ANCOVA) with treatment as the factor and the baseline value (in the case of 11-Point Box Scales: average value of the week until baseline) of the respective effectiveness variable as a covariate. The confidence intervals of the differences in the least square means (LS means) were computed to compare the effectiveness of the treatments. For the HADS and the SDS changes from baseline were compared between the EGb 761^®^ group and the pentoxifylline group by the Wilcoxon rank sum test. Changes for ordinal variables over time were modelled with generalized estimating equations (GEEs) for ordinal responses.

Descriptive statistics were computed to describe the empirical distributions; 95% confidence intervals were calculated within the treatment groups and between the pentoxifylline and the EGb 761^®^ group. Since no confirmatory hypotheses were formulated, an adjustment of the type-one error rate was not performed and no formal sample size calculation was done. All *p* values presented are two-sided and should be interpreted in an exploratory sense. The presented results are based on the full analysis set (FAS) using the last observation carried forward method (LOCF) to replace missing values. Furthermore, all analyses were also performed based on observed cases (OC). The results based on the OC were generally very consistent with those for the FAS and are therefore not presented.

## Results

### Participant flow and treatment compliance

Patients were recruited in one investigational centre from September 2012 to April 2014. In total, 202 subjects were screened for inclusion in the study, 2 subjects terminated the study before the baseline visit and were not randomized. All remaining 200 subjects were included in the double-blind treatment phase; they were randomized to and received EGb 761^®^ (n = 100) or pentoxifylline (n = 100).

**Fig. 1:**
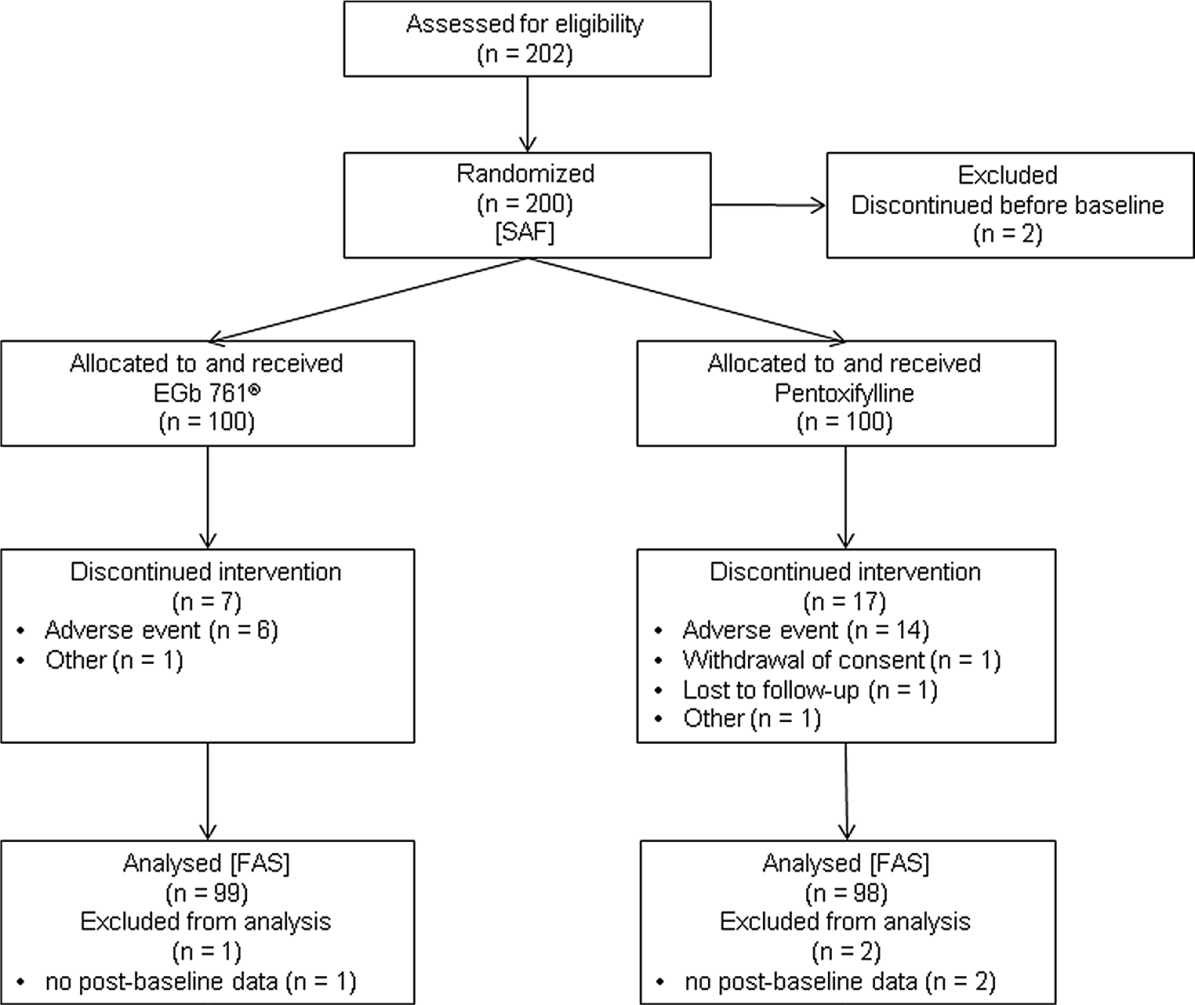
Patient disposition

All subjects who received at least one dose of the treatment were analysed with regard to safety measures (safety analysis set, SAF). The full analysis set (FAS) for efficacy included all subjects of the SAF having at least one post-baseline measurement of one of the 11-Point Box Scales. The disposition of patients is depicted in Fig. 1.

### Demographics and baseline characteristics

Both treatment groups were similar with respect to demographics and baseline scores of the outcome measures (Table 1). All subjects were white/caucasian.

**Table 1:** Demographics and baseline characteristics; absolute (relative) frequency or mean ± SD [95% CI for mean]

	EGb 761^®^ (n = 99)	Pentoxifylline (n = 98)
Women	58 (58.6%)	59 (60.2%)
Age (years)	55.4 ± 10.5	53.1 ± 10.9
	[53.3; 57.5]	[50.9; 55.3]
Weight (kg)	82.6 ± 16.2	77.8 ± 13.7
	[79.4; 85.8]	[75.0; 80.5]
BMI (kg/m^2^)	27.2 ± 4.4	25.9 ± 3.5
	[26.3; 28.0]	[25.2; 26.5]
Duration of tinnitus (months)	79.6 ± 77.9	84.6 ± 94.5
	[64.0; 95.1]	[65.7; 103.6]
Patients with tinnitus > 2 years	74 (74.7%)	75 (76.5%)
Patients with hearing loss	97 (98.0%)	92 (93.9%)
Mini-TQ	10.5 ± 4.3	11.0 ± 4.3
	[9.62; 11.35]	[10.12; 11.84]
11-Point Box Scale loudness	5.3 ± 1.3	5.6 ± 1.2
	[4.99; 5.52]	[5.37; 5.87]
11-Point Box Scale annoyance	5.2 ± 1.3	5.5 ± 1.3
	[4.95; 5.46]	[5.30; 5.80]
HADS anxiety score	6.2 ± 3.3	5.9 ± 3.5
	[5.53; 6.86]	[5.23; 6.61]
HADS depression score	4.7 ± 3.1	4.4 ± 3.0
	[3.94; 5.26]	[3.40; 4.79]
SDS global impairment	7.5 ± 5.7	8.9 ± 5.9
	[6.31; 8.61]	[7.72; 10.11]

The assessment of compliance was based on the difference between the numbers of tablets dispensed and returned, expressed as percentage of tablets due to be taken from the day of first to the day of last intake. Median compliance was 99.4% for the total treatment period in the EGb 761^®^ group and 98.8% in the pentoxifylline group.

### Therapeutic effects

Patients of both treatment groups improved significantly during the 12-week treatment period on all tinnitus-related scales, in anxiety and disability scores, with no significant differences between the two treatment groups. Details are shown in Tables 2 and 3.

**Table 2:** Changes from baseline to week 12 in tinnitus-related outcomes; least square mean (95% CI) and *p* values from ANCOVA for within- and between-group comparisons

	EGb 761^®^	*p* value (within-group)	Pentoxifylline	*p* value (within-group)	*p* value (between-group)
Mini-TQ	− 1.57 (− 2.25; − 0.89)	< 0.0001	− 1.94 (− 2.64; − 1.25)	< 0.0001	0.4514
11-Point Box Scale loudness	− 0.41 (− 0.68; − 0.15)	0.0021	− 0.43 (− 0.69; − 0.17)	0.0015	0.9284
11-Point Box Scale annoyance	− 0.56 (− 0.84; − 0.27)	0.0002	− 0.54 (− 0.83; − 0.25)	0.0003	0.938

**Table 3:** Changes from baseline to week 12 in affective symptoms and disability; mean (95% CI) and two-sided *p* values from Wilcoxon rank sum test for between-group comparisons

	EGb 761^®^	Pentoxifylline	*p* value
HADS anxiety score	− 1.3 (− 1.82; − 0.85)	− 1.1 (− 1.55; − 0.56)	0.2523
HADS depression score	− 0.4 (− 0.89; 0.15)	− 0.5 (− 0.92; 0.01)	0.5753
SDS global impairment	− 0.6 (− 0.90; − 0.28)	− 0.6 (− 0.91; − 0.27)	0.9485

Slight improvements in the depression score of the HADS were not statistically significant at 12 weeks. However, in the prospectively specified subgroup of patients with elevated depression scores (HADS subscore depression ≥ 8, with 8 to 10 points representing borderline depression and 11 points or more indicating clinical caseness [12]), the improvements on the three tinnitus-related scales were particularly pronounced under EGb 761^®^ treatment (Mini-TQ: − 2.19 [− 3.96; − 0.42]; 11-Point Box Scale loudness: − 0.74 [− 1.45; − 0.02]; 11-Point Box Scale annoyance: − 1.06 [− 1.93; − 0.18]). Such an effect modification by depression was not seen in the pentoxifylline group.

**Fig. 2:**
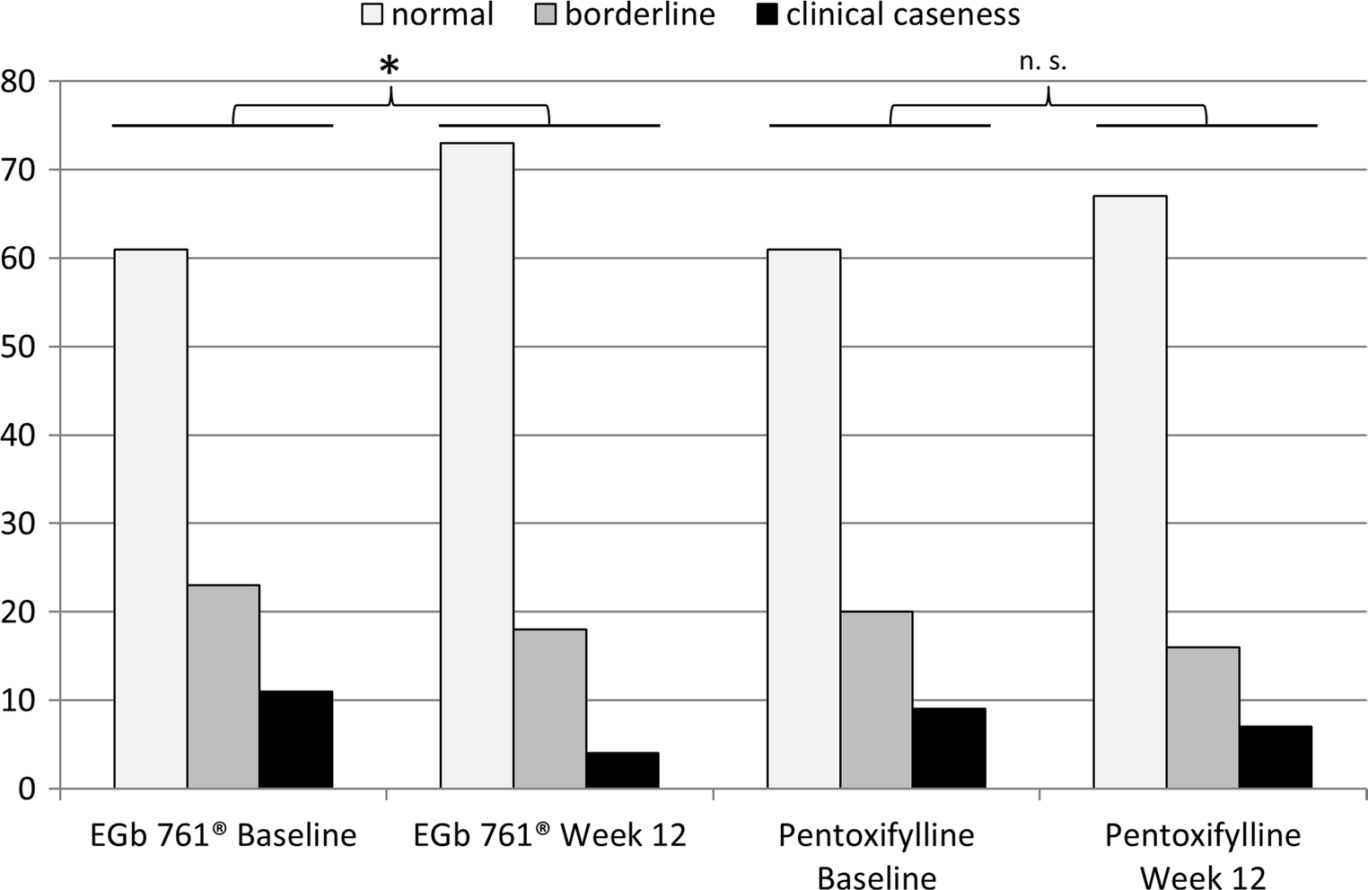
Numbers of patients with normal and abnormal scores at baseline and week 12 in the HADS anxiety subscale (scores of 0–7 are considered normal, 8–10 borderline, and ≥ 11 indicate clinical caseness); *two-sided *p* value for likelihood score test for changes over time (modelled by GEEs for ordinal responses) = 0.005 for EGb 761^®^ (non-significant (n.s.) at *p* = 0.105 for pentoxifylline)

Of 95 patients in the EGb 761^®^ group and 90 patients in the pentoxifylline group who had HADS anxiety scores before and after treatment, 34 (36%) and 29 (32%), respectively, had abnormal scores at baseline. These numbers decreased to 22 (23%) and 23 (26%), respectively, during treatment (Fig. 2). The changes were significant in the EGb 761^®^ group, but not in the pentoxifylline group (*p* = 0.005 and *p* = 0.105, respectively, two-sided likelihood score test for changes over time modelled by GEEs for ordinal responses).

### Safety and tolerability

During the active treatment and subsequent 2-day risk phase (i.e. until active substances were washed out), 19/100 (19.0%) subjects in the EGb 761^®^ group experienced a total of 20 AEs leading to an overall incidence rate of 0.0024 AEs/day of exposure. In the pentoxifylline group, 27/100 (27.0%) subjects experienced a total of 36 AEs leading to an overall incidence rate of 0.0048 AEs/day of exposure. Therefore, in the pentoxifylline group the incidence rate for AEs was twice as high as in the EGb 761^®^ group. A causal relationship with the investigational product could not be excluded for 18 AEs in 17/100 (17%) subjects in the EGb 761^®^ group, in the pentoxifylline group, a causal relationship with the investigational product could not be excluded for 32 AEs in 24/100 (24%) subjects. No serious adverse event was reported during the study. The most frequent AEs are listed in Table 4. No clinically relevant changes regarding mean values of laboratory parameters (haematology, blood chemistry including liver enzymes and coagulation parameters), physical examination, blood pressure, heart rate or weight were observed between screening and end of treatment. In two subjects of the EGb 761^®^ group relevant changes were observed with regard to the 12-lead ECG between screening and end of treatment. In both cases, the causal relationship was assessed as unlikely.

**Table 4:** Most frequently reported adverse events (5% or more) under EGb 761^®^ and pentoxifylline treatment, respectively

System organ class/event	EGb 761^®^	Pentoxifylline
Gastrointestinal disorders	2	11
Diarrhoea	2	
Abdominal discomfort		1
Abdominal distension		2
Upper abdominal pain		4
Dyspepsia		1
Nausea		2
Unspecified symptom		1
Ear and labyrinth disorders	6	4
Worsening of tinnitus	5	3
Ear discomfort	1	
Vertigo		1
Infections and infestations	2	5
Bronchitis	1	1
Nasopharyngitis	1	2
Gastroenteritis		1
Pertussis		1

## Discussion

The objective of this randomized, double-blind, reference-controlled single-centre trial was to compare the treatment effects of Ginkgo biloba extract EGb 761^®^ and pentoxifylline in subjects with sub-chronic or chronic tinnitus focusing on psycho-social problems. We found significant improvements under both treatments on a self-rating scale assessing the psychological burden of tinnitus (Mini-TQ), on numeric analogue scales (11-Point Box Scales) for tinnitus loudness and annoyance, the anxiety subscale of a questionnaire for anxiety and depression (HADS) and a brief rating of illness-related disability.

The treatment of chronic tinnitus is a very challenging task in everyday practice, especially due to its various origins. Tinnitus itself cannot be considered as a disease but rather as a symptom. The pharmacological or non-pharmacological therapeutic approach should take the suspected possible cause in each individual case into consideration. This is influenced by the patient’s medical history, risk factors, concomitant diseases and precipitating events. The aetiology might be otological, neurological, metabolic, cardiovascular, endocrinological, musculoskeletal or mental. The use of EGb 761^®^ and pentoxifylline in this trial was based on the fact that both drugs are frequently used in many European countries [15]. Perfusion-enhancing properties that act in the brain and inner ear [16–18] are assumed to contribute to the clinical benefits.

Despite the efforts of strict categorization, tinnitus changes and modulates with time. The physicians therefore have to cope with the central processing of tinnitus as well as the various psychological reactions of the patients to the tinnitus. Psychological and social aspects of tinnitus can severely affect patients’ quality of life. The evaluation of the treatment effects was therefore extended from single subjective perception of the ear sound to include anxiety, depression and overall disability symptoms of the subjects. In this respect, the anxiolytic [19] and antidepressant-like effects [20, 21] of EGb 761^®^, as well as its influence on neuroplasticity, involving neurogenesis and synaptogenesis [22], may play a role.

The majority of the study patients had been suffering from chronic tinnitus for many months (average duration 7 years) and had already undergone many therapeutic procedures. Only very few (7/197) were treated with any medication shortly before enrolment into the study. Hence, the study population appears to represent those patients who are most difficult to manage [23, 24]. In view of this, the statistically significant decrease in subjective perception of tinnitus (Mini-TQ, 11-Point Box Scales for loudness and annoyance) points to benefits of both tested medications. Patients with tinnitus often have sub-syndromal depression or anxiety, which may be reactive in nature or due to a common organic origin [6]. It is therefore noteworthy that EGb 761^®^ has stronger effects in patients with depression and decreases anxiety levels.

The relatively large sample size and the high treatment adherence may be considered as strengths of this trial; limitations are the reliance on tablet count for the determination of adherence and the monocentric setting. The present results are in line with and extend earlier findings that demonstrated the efficacy of both drugs in the treatment of tinnitus [25–27]. It adds to current knowledge the direct comparison of the two drugs in terms of efficacy and tolerability. There is no evident superiority of one investigational product over another in terms of efficacy; however, tolerability of EGb 761^®^ is clearly better. During the study period, the total numbers of AEs differed considerably. In the EGb 761^®^ group the risk of an adverse event was about half the risk observed in the pentoxifylline group.

Further studies to disentangle the direct effects of EGb 761^®^ on tinnitus and presumable indirect effects that may result from improvements in anxiety and depression are warranted.

## Conclusion

In summary, the two drugs, EGb 761^®^ and pentoxifylline are similarly effective in reducing the suffering of patients with sub-chronic or chronic tinnitus. The EGb 761^®^ treatment group showed a more pronounced improvement in patients with elevated depression scores and a higher incidence of improved patients in anxiety score categories. The incidence of ADEs was clearly lower in the EGb 761^®^ group.

## Footnotes

1 Manufacturer: Dr. Willmar Schwabe GmbH & Co. KG, Karlsruhe, Germany. EGb 761^®^ is a registered trademark of Dr. Willmar Schwabe GmbH & Co. KG.
