# Correction to: Ginkgo biloba extract EGb 761^®^ versus pentoxifylline in chronic tinnitus: a randomized, double-blind clinical trial

**DOI:** 10.1007/s11096-018-0704-y

**Published:** 2018-08-28

**Authors:** Klára Procházková, Ivan Šejna, Jan Skutil, Aleš Hahn

**Affiliations:** 1 Department of Otorhinolaryngology, University Hospital Kralovske Vinohrady, Šrobárova 50, 10034 Prague, Czech Republic; 2 Third Faculty of Medicine, Charles University, Prague, Czech Republic

## Correction to: International Journal of Clinical Pharmacy 10.1007/s11096-018-0654-4

The article Ginkgo biloba extract EGb 761^®^ versus pentoxifylline in chronic tinnitus: a randomized, double-blind clinical trial, written by Klára Procházková, Ivan Šejna, Jan Skutil and Aleš Hahn, was originally published electronically on the publisher’s internet portal (currently SpringerLink) on 01 June 2018 without open access.

With the author(s)’ decision to opt for Open Choice the copyright of the article changed on July 2018 to © The Author(s) 2018 and the article is forthwith distributed under the terms of the Creative Commons Attribution 4.0 International License (http://creativecommons.org/licenses/by/4.0/), which permits use, duplication, adaptation, distribution and reproduction in any medium or format, as long as you give appropriate credit to the original author(s) and the source, provide a link to the Creative Commons license and indicate if changes were made.

